# Biomechanical Fatigue Behavior of a Dental Implant Due to Chewing Forces: A Finite Element Analysis

**DOI:** 10.3390/ma17071669

**Published:** 2024-04-05

**Authors:** Miguel Martinez-Mondragon, Guillermo Urriolagoitia-Sosa, Beatriz Romero-Ángeles, Miguel Angel García-Laguna, Aldo Saul Laguna-Canales, Juan Carlos Pérez-Partida, Jonatan Mireles-Hernández, Francisco Carrasco-Hernández, Guillermo Manuel Urriolagoitia-Calderón

**Affiliations:** 1 Instituto Politécnico Nacional, Escuela Superior de Ingeniería Mecánica y Eléctrica, Sección de Estudios de Posgrado e Investigación, Unidad Profesional Adolfo López Mateos Zacatenco, Edificio 5, 2do, Piso, Col. Lindavista, Del. Gustavo A. Madero, Ciudad de México C.P. 07320, Mexico; romerobeatriz97@hotmail.com (B.R.-Á.);; 2 Universidad Abierta y a Distancia de México, División de Ciencias de la Salud, Biológicas y Ambientales, Av. Universidad 1200, Piso 1, Cuadrante 10, 1-2, Xoco, Alcaldía Benito Juárez, Ciudad de México C.P. 03330, Mexico; 3 Universidad Tecnológica de Durango, Mecatrónica y Energías Renovables, Carretera Durango-Mezquital, km 4.5 S/N, Gavino Santillán, Durango C.P. 34308, Mexico

**Keywords:** dental implant, abutment, biomechanics, biomaterials, PEEK, titanium, fatigue, simulation

## Abstract

The use of titanium as a biomaterial for the treatment of dental implants has been successful and has become the most viable and common option. However, in the last three decades, new alternatives have emerged, such as polymers that could replace metallic materials. The aim of this research work is to demonstrate the structural effects caused by the fatigue phenomenon and the comparison with polymeric materials that may be biomechanically viable by reducing the stress shielding effect at the bone–implant interface. A numerical simulation was performed using the finite element method. Variables such as Young’s modulus, Poisson’s coefficient, density, yield strength, ultimate strength, and the S-N curve were included. Prior to the simulation, a representative digital model of both a dental implant and the bone was developed. A maximum load of 550 N was applied, and the analysis was considered linear, homogeneous, and isotropic. The results obtained allowed us to observe the mechanical behavior of the dental implant by means of displacements and von Mises forces. They also show the critical areas where the implant tends to fail due to fatigue. Finally, this type of non-destructive analysis proves to be versatile, avoids experimentation on people and/or animals, and reduces costs, and the iteration is unlimited in evaluating various structural parameters (geometry, materials, properties, etc.).

## 1. Introduction

In the last 20 years, dental implants have been proven and have become treatments with a high success rate, achieving effectiveness of more than 90% when replacing one or several teeth [1,2]. In addition to its long life, restoring function, and recovering oral aesthetics, modern science has allowed for constant growth, taking advantage of new technologies that evolved the field of dentistry [3,4]. Despite this, and regardless of preventive measures, the possibility of having complications and being able to suffer a biological and/or mechanical failure is not ruled out. A very common biological failure is marginal bone loss that can be caused due to surgical trauma, excessive occlusal load, microbial contamination, micro-movements between implant and abutment, etc. [5,6]. On the other hand, mechanical failures involve fractures due to structural problems in the dental implant caused by occlusal overload, material fatigue, poor design, and even improper placement of the dental implant [7,8,9].

Titanium and its alloys have been the most widely used and successful materials in dentistry and orthopedics for many years. When applying a dental implant treatment, 95% of the material of choice is titanium due to the characteristics that favor it such as the following: good resistance, rigidity, ductility, corrosion resistance, and its high biocompatibility with the oral environment for excellent osseointegration [10,11]. That said, methodologies and processes for the development of new biomaterials are of great importance and vital for a continuous advance in dental applications. Therefore, dental biomaterials must not only have the ability to withstand the efforts generated by masticatory forces but must also be as immune as possible to substances that can occur in the mouth, as well as to ingested food, in order to reduce an adverse biological response (susceptible to corrosion and ions released) during the life of the dental implant [12,13,14].

Titanium has proven to be a material with excellent physical and mechanical characteristics to be used in dental implants; however, its high rigidity (110 MPa) compared with that of bone (3–30 MPa) does not allow for efficiency in the distribution of forces (stress shielding effect), so the load-induced stimulation between the bone–implant interface is minimal, resulting in a gradual bone loss [15,16,17]. Not to mention clinical problems derived from the use of titanium such as disseminated radiation, surface degeneration, peri-implantitis, sensitivity, allergies, and reduced image quality in imaging studies (radiographs) that affect the evaluations of pathological conditions, causing poor diagnosis [18,19]. In addition to this, an alternative biomaterial for dental implants that has been studied in recent decades is polymers due to their characteristics that integrate excellent physical and mechanical properties, as well as good biocompatibility when applying treatments or surface coatings [20,21,22]. Among those that stand out are polyetherectone (PEKK) and polyetheretherketone (PEEK), which are the best-known of the polyaryleketone (PAEK) family. PEEK is a biphasic semi-crystalline polymer that emerged in the late 1990s and boasts excellent biological, mechanical, and physical properties for biomedical applications [23,24,25,26]. PEEK has been studied and used for over fifteen years as a biomaterial to replace parts of the spine, hip prostheses, and cranial cortices, making it a great alternative for dental implants. Compared with metals, PEEK is non-corrosive, minimizes metal allergies, and has a low specific gravity relative to strength [27,28,29,30]. In addition to its high mechanical and chemical resistance, it has a low elastic modulus (3–4 MPa) that, combined with other materials such as carbon fiber, allows the elastic modulus to be increased to 18 MPa, a value closer to human bone [31,32]. This feature makes it a promising biomaterial that could reduce the stress shielding effect, which is a very common problem when it comes to endosseous implants. This effect is caused by an insufficient load distribution between bone and an implant, causing a decrease in tissue mass and, therefore, bone resorption. As is the case with Ti, only a part of the load is transferred to the bone and most falls on the implant. This effect can be corrected either by means of structures with a better geometric design or new biomaterials with better biomechanical properties that allow them to increase their useful life [33,34,35].

Chewing is a complex biomechanical process involving several structures including bones, muscles, joints, and teeth. This process involves the movement of the jaw when food is inside the mouth, initiating the chewing cycle [36]. Therefore, dental implants are subject to a large number of load cycles during their useful life. This causes mechanical wear of the material (fatigue phenomenon), which decreases its resistance, making it prone to the initiation of microcracks and resulting in the propagation of this until it reaches breakage [37,38]. An important aspect of the success or failure of a dental implant is the way in which the forces caused by masticatory forces are transferred to the surrounding bone. Therefore, it is important to perform a stress analysis of the bone–implant interface to understand the impact with respect to the strength of the material and the incidence of failures, where an overload would cause bone resorption or failure due to implant fatigue, while insufficient bone loading would cause atrophy due to disuse and, therefore, bone loss [39,40,41,42].

On the other hand, the use of numerical techniques such as the finite element method allows for the analysis of a variety of physical phenomena. This technique is based on the representation of mathematical models that allow for understanding and quantifying the results obtained under a good interpretation of physical phenomena. This has made the finite element method a versatile and functional tool to obtain rapid results, which has recently had an increase in use because of technological development and its application in various lines of research [43,44]. Such is the case for the medical field, where it is extremely useful for the study of complex anatomical systems that are often difficult to analyze in vivo or in vitro. Likewise, biomechanical behaviors that include some stimulation, pathologies, or trauma can be analyzed [45]. In dentistry, it is very common for the finite element method to be used to evaluate the distribution of forces at the bone–implant interface, in prosthetic components, dental wear, etc. [46,47], or, in the case of orthodontics, is it used to evaluate the mechanical behavior of the periodontal ligament, malocclusions, root fracture, etc. Broadly speaking, for the study of the mechanics of tooth movement, this non-invasive analysis technique offers the ease of saving computational resources and time with unlimited use of iterations, making it efficient and cost-effective [48,49,50].

The purpose of this research work is to demonstrate that polymeric materials can be a viable option to replace titanium in dental applications. Because most of this research presents cases of static analysis involving a single loading condition, a numerical evaluation is carried out that includes repetitive loads assimilating the function of chewing. In this way, it is possible to determine the biomechanical behavior that is manifested in the structure of the dental implant due to the phenomenon of fatigue.

## 2. Materials and Methods

The development of the numerical analysis was carried out based on the ISO 14801 standard (UNE-ISO 14801:2017), taking as a reference the parameters and conditions that it describes for an experimental fatigue test in dental implants in unfavorable conditions such as bone recession. It was decided to carry out this research numerically and not experimentally because, on the one hand, of the cost and time it takes to characterize the material and perform the tests. On the other hand, applying computational resources such as the use of the finite element method (FEM), which is based on algorithmic interpretations through software such as Ansys (R2 student version 2022), Abacus, SolidWorks (student version 2022), etc., is effective and maintains an assertiveness of 97%. In addition, there is already research in the literature on the properties of materials.

### 2.1. Characteristics of the Dental Implant

**Figure 1:**
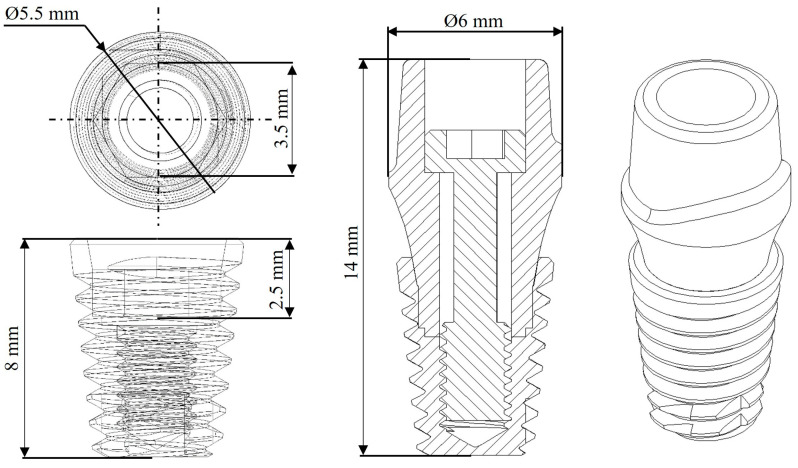
Dental implant dimensions.

The characteristics of the implant were taken based on the anatomy of a lower first molar, so the dimensions were considered and compared according to catalogs of commercial manufacturers (Straumann (Basel, Switzerland), Avinent (Santpedor, Spain), Nobel Biocare (Kloten, Switzerland)). Therefore, and according to the proposal, a two-piece dental implant was selected with an internal conical hexagonal connection joined by a screw. The dimensions of the dental implant are 14 mm in length and a diameter of 6 mm (Figure 1). In addition to this, and according to the research literature, the average chewing force of a human being is 300–450 N, and the maximum that can be reached in healthy conditions is 450–550 N, with bruxism up to 850 N [28,51,52].

### 2.2. Dental Implant Modeling

**Figure 2:**
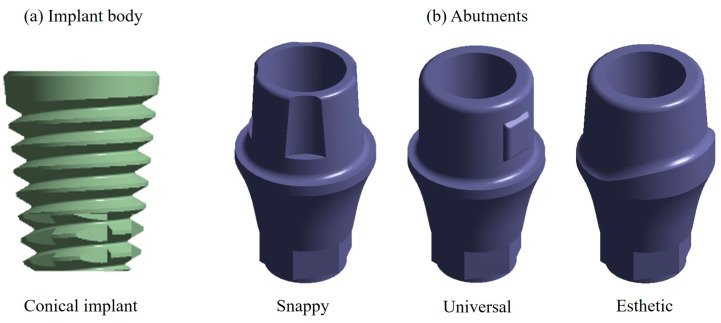
(**a**) Conical implant. (**b**) Abutments including Snappy, Universal, and Esthetic.

The model of the dental implant was developed using the SolidWorks computer program. For the modeling of the dental implant, designs of commercial manufacturers were used. In the case of the dental implant body, a conical geometry was chosen (Figure 2a) because of a better grip; for the abutment, three different designs were chosen, which were all joined by a prosthetic screw developed by the Swiss company Nobel Biocare (Kloten, Switzerland), called Snappy abutment, Universal and Esthetic (Figure 2b).

### 2.3. Material Properties

Several materials are characterized as being biocompatible with the human body; however, when it comes to treatments with dental implants, metallic materials predominate, specifically titanium and its alloys [53]. To this end, two case studies were carried out as follows: in the first case, titanium with an alpha–beta composition (Ti_6_Al_4_V Grade 5) was used, and, for the second case, a polymeric material was chosen, which was carbon fiber-reinforced polyether–ether–ketone (CFR-PEEK) [54,55]. Finally, in both cases, a spherical member was added in the upper area of the abutment, as marked by the norm simulating the prosthesis (dental crown), where, for this case, zirconium (ZrO_2_) was used as the material. The mechanical properties can be seen in Table 1 [56,57].

### 2.4. Boundary and Loading Conditions

**Figure 3:**
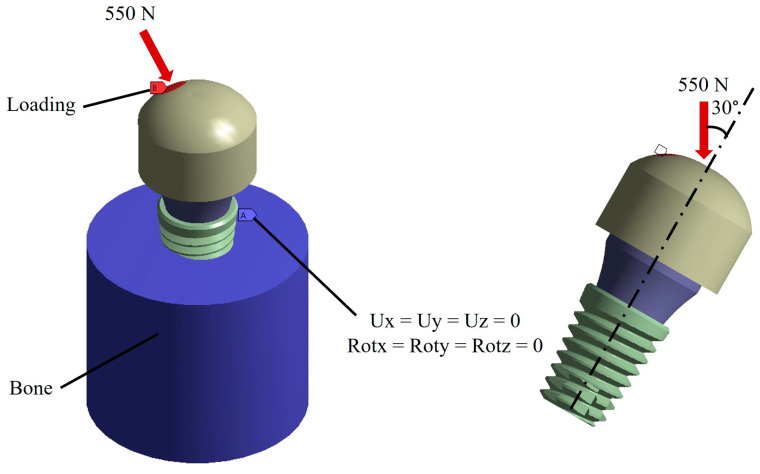
Boundary conditions and external load.

According to the anatomy of the tooth, the boundary conditions were placed in the bone zone restricting the six degrees of freedom including displacements in Ux, Uy, and Uz and rotations in Rx, Ry, and Rz. On the other hand, the loading conditions were placed in the upper part of the spherical member, and the direction of the load was placed based on the ISO 14801 standard that mentioned applying the load at an angle of 30° with respect to the vertical of the implant. For the magnitude, the maximum chewing force of a healthy person was taken as a reference, which is 550 N (Figure 3) [58,59].

### 2.5. Analysis

**Figure 4:**
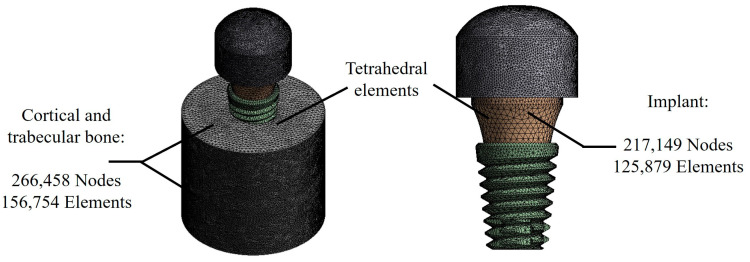
Discretizing the complete model.

The models presented above were evaluated by means of the finite element method using ANSYS Workbench (ANSYS, Inc., San Diego, CA, USA) as a computer program. For the analysis, a fine discretized with tetrahedral elements and a maximum element size of 0.2 mm was used, giving an average total of 483,607 nodes and 282,633 elements among the three different models (Figure 4). The behavior of the dental implant model was considered linear, homogeneous, and isotropic, although it should be clarified that the behavior of the cortical and trabecular bone area was considered with orthotropic properties.

## 3. Results

**Figure A1:**
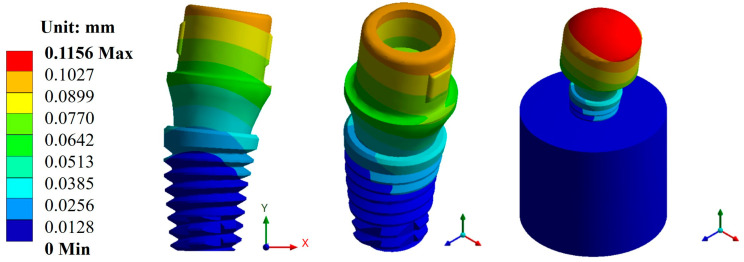
Total general displacement for the Universal abutment type (Ti_6_Al_4_V).

**Figure A2:**
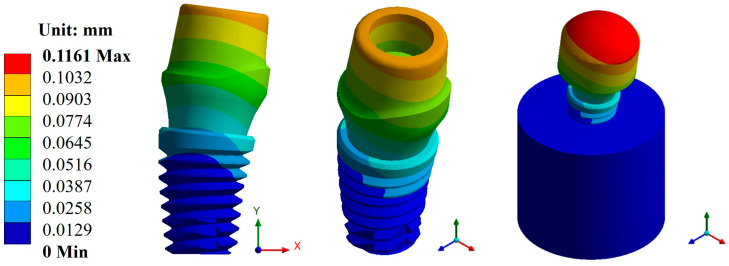
Total general displacement for the Esthetic abutment type (Ti_6_Al_4_V).

**Figure A3:**
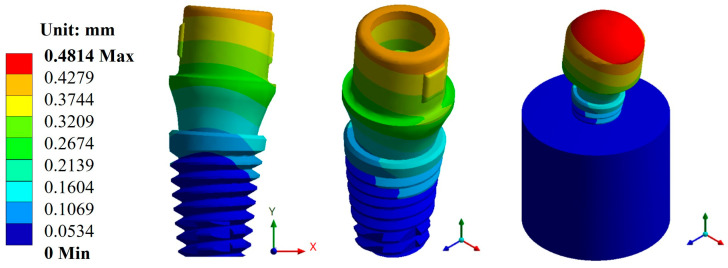
Total general displacement for the Universal abutment type (CFR-PEEK).

**Figure A4:**
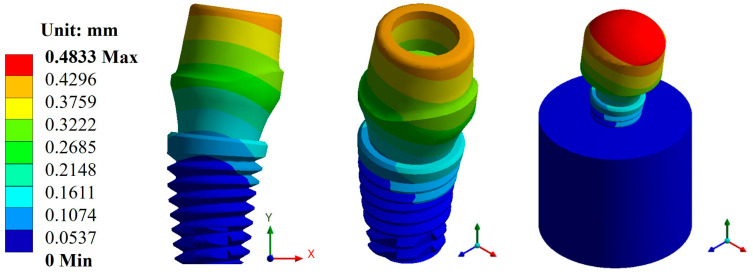
Total general displacement for the Esthetic abutment type (CFR-PEEK).

**Figure A5:**
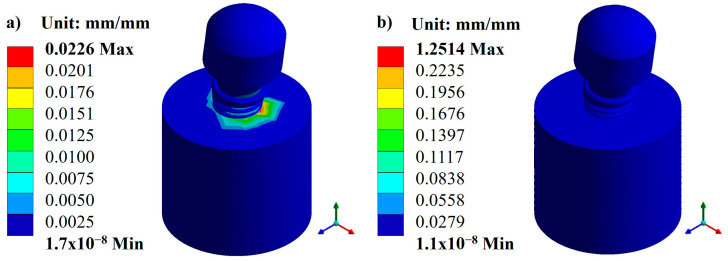
Total unit deformation for the Universal abutment: (**a**) titanium and (**b**) CFR-PEEK.

**Figure A6:**
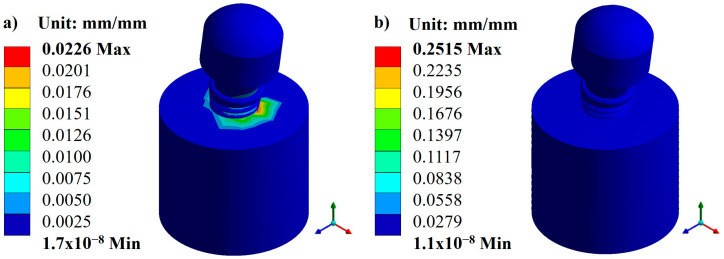
Total unit deformation for the Esthetic abutment: (**a**) titanium and (**b**) CFR-PEEK.

**Figure A7:**
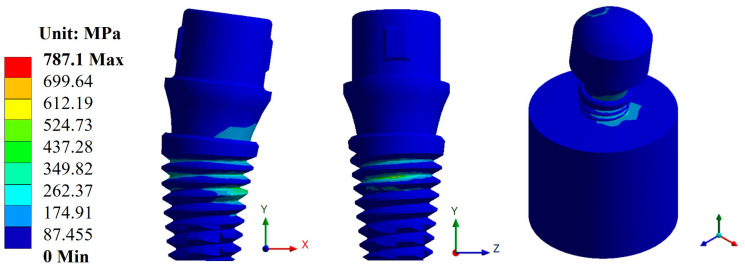
von Mises stress for the Universal abutment type (Ti_6_Al_4_V).

**Figure A8:**
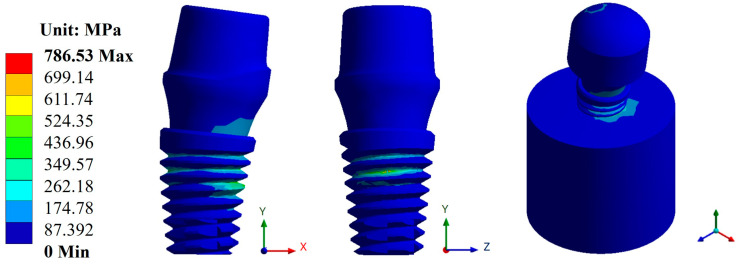
von Mises stress for the Esthetic abutment type (Ti_6_Al_4_V).

**Figure A9:**
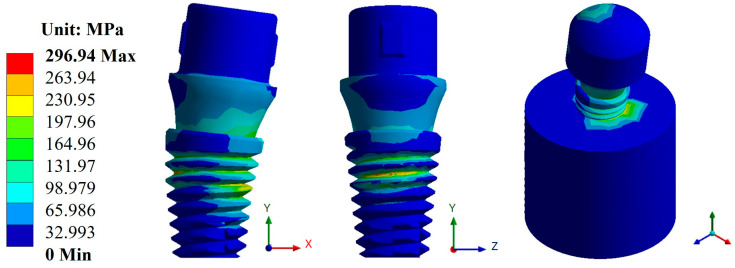
von Mises stress for the Universal abutment type (CFR-PEEK).

**Figure A10:**
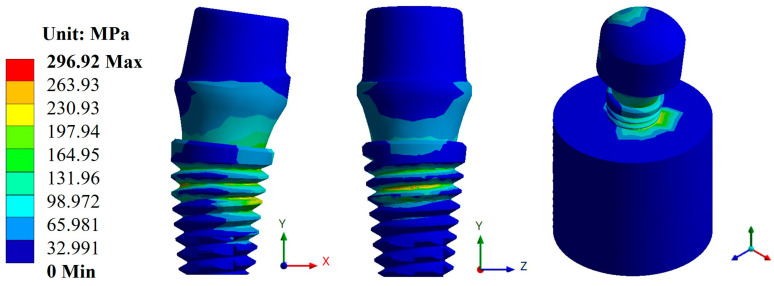
von Mises stress for the Esthetic abutment type (CFR-PEEK).

**Figure A11:**
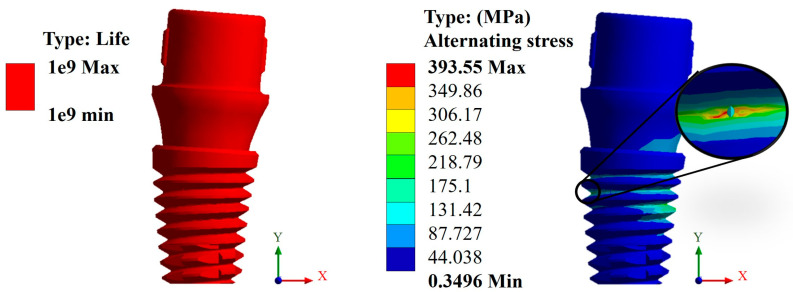
Life cycles and fatigue alternating stress for the Universal abutment type (Ti_6_Al_4_V).

**Figure A12:**
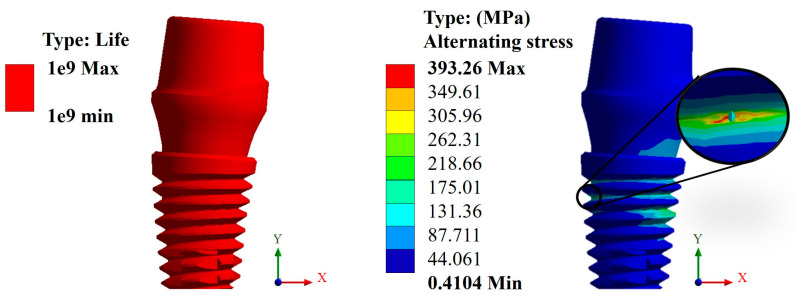
Life cycles and fatigue alternating stress for the Esthetic abutment type (Ti_6_Al_4_V).

**Figure A13:**
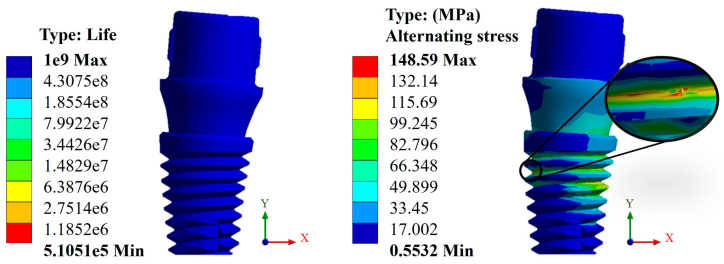
Life cycles and fatigue alternating stress for the Universal abutment type (CFR-PEEK).

**Figure A14:**
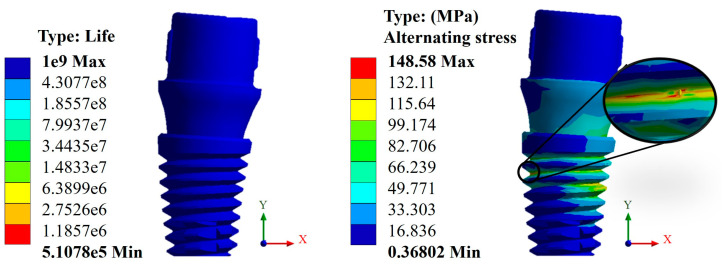
Life cycles and fatigue alternating stress for the Esthetic abutment type (CFR-PEEK).

The results of the numerical analysis are presented below, mainly highlighting the global total displacements, unit deformations, stresses by von Mises failure criterion, and life cycles due to fatigue for each abutment and for both materials. It is worth mentioning that the results presented in this section are for the most optimal pillar; the results of the other pillars can be observed in Appendix A (Figure A1, Figure A2, Figure A3, Figure A4, Figure A5, Figure A6, Figure A7, Figure A8, Figure A9, Figure A10, Figure A11, Figure A12, Figure A13 and Figure A14). Finally, at the end of this section, there is a table with all the results obtained (Table 2).

### 3.1. Displacements and Deformations

**Figure 5:**
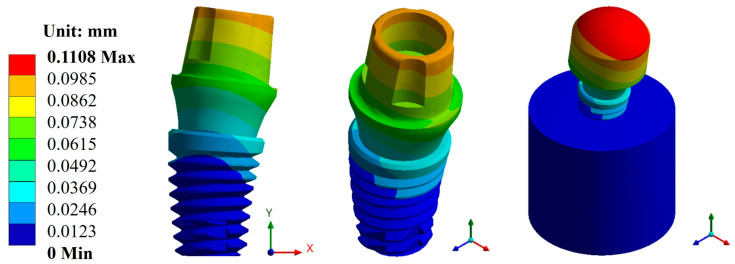
Total general displacement for the Snappy abutment type (Ti_6_Al_4_V).

**Figure 6:**
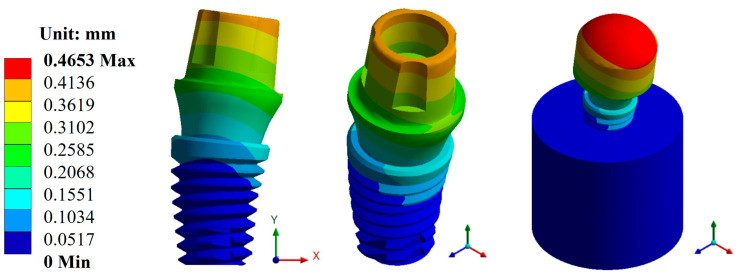
Total general displacement for the Snappy abutment type (CFR-PEEK).

**Figure 7:**
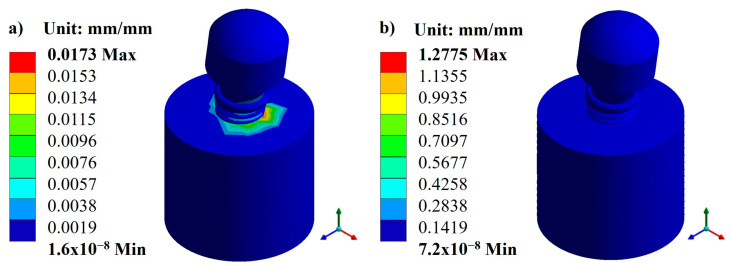
Total unit deformation for the Snappy abutment: (**a**) titanium and (**b**) CFR-PEEK.

**Figure 8:**
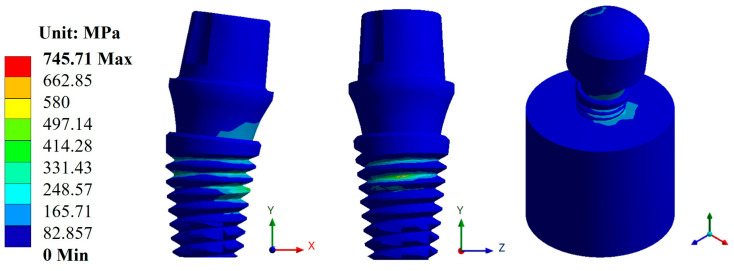
von Mises stress for the Snappy abutment type (Ti_6_Al_4_V).

Based on the characteristics and parameters applied, the biomaterial with the lowest displacement was titanium, with an average value of 0.1141 mm compared with CFR-PEEK, which had an average value of 0.4766 mm. On the other hand, the three pillars showed a displacement with a minimal difference. However, the Snappy pillar showed better mechanical behavior in both materials (Figure 5 and Figure 6). In addition to the above, Figure 7 and Figure 8 show the total unit deformation of both biomaterials, which indicates the area where it is distorted or undergoes a change in geometry derived from the applied load.

### 3.2. von Mises Stress

**Figure 9:**
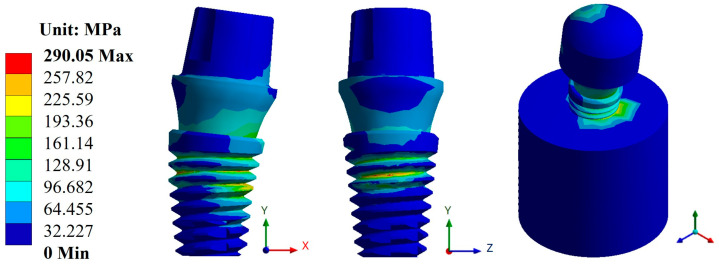
von Mises stress for the Snappy abutment type (CFR-PEEK).

In the case of stresses, the biomaterial CFR-PEEK showed the best result with an average value of 294.63 MPa compared with titanium, which had an average value of 773.11 MPa. Likewise, from the evaluation of the three dental abutments, the best result was again in the Snappy abutment with a maximum value of 290.05 MPa in CFR-PEEK and a value of 745.71 MPa in titanium (Figure 8 and Figure 9). In the figures mentioned above, you can see the areas where there is a greater concentration of stress both in the implant and in the bone, and this indicates the most susceptible areas where the material can fail.

### 3.3. Life Cycles to Fatigue

**Figure 10:**
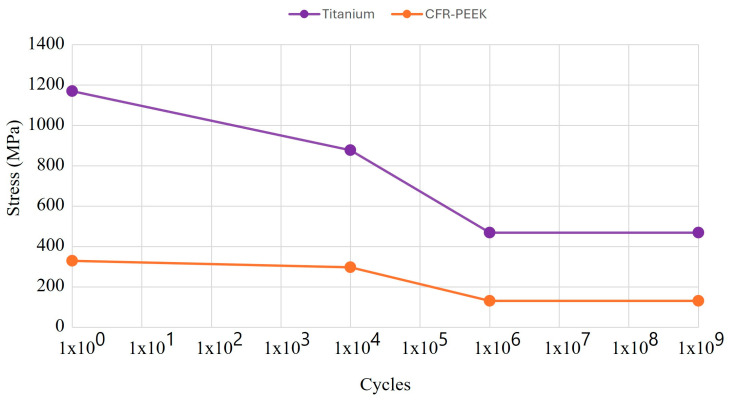
S-N curve of fatigue behavior of titanium and CFR-PEEK.

For the fatigue analysis, the S-N curve, also known as the Wöhler curve, was used, which is a graph that represents the fatigue behavior of a material, where the number of cycles is on the abscissa axis and stress is on the ordinate axis, both generally on a logarithmic scale (Figure 10) [60,61]. In addition, two parameters that the authors consider relevant were taken into account for the results. One is the life cycle, which indicates the failure of the material due to repeated loads (the useful life of the material). The other is alternating stress, which is a relationship between the maximum and minimum effort that acts on the dental implant and works as a metric with respect to the fatigue limit of the material, so it must be kept in the high-cycle zone to avoid failure.

**Figure 11:**
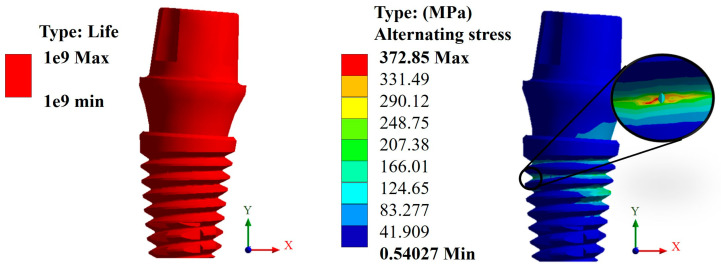
Life cycles and fatigue alternating stress for the Snappy abutment (Ti_6_Al_4_V).

**Figure 12:**
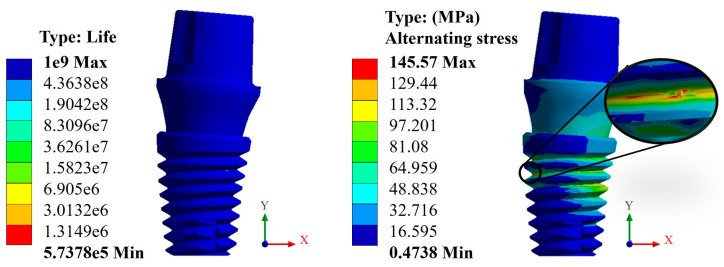
Life cycles and fatigue alternating stress for the Snappy abutment (CFR-PEEK).

In this case, again, the Snappy pillar showed a better geometric behavior. In addition, the titanium biomaterial showed a better mechanical behavior compared with CFR-PEEK, as expected due to its high rigidity, giving a favorable result corresponding to over one million load cycles. This can be verified with the alternating stress that resulted in a maximum value of 372.85 MPa, which is less than the fatigue limit of 468 MPa (Figure 11). In the case of CFR-PEEK, certain areas did not reach values above one million load cycles, as it was intended, obtaining a maximum value of 573,780 cycles with a maximum alternating stress value of 145.57 MPa, which is higher than the fatigue limit of the biomaterial of 132 MPa (Figure 12).

**Figure 13:**
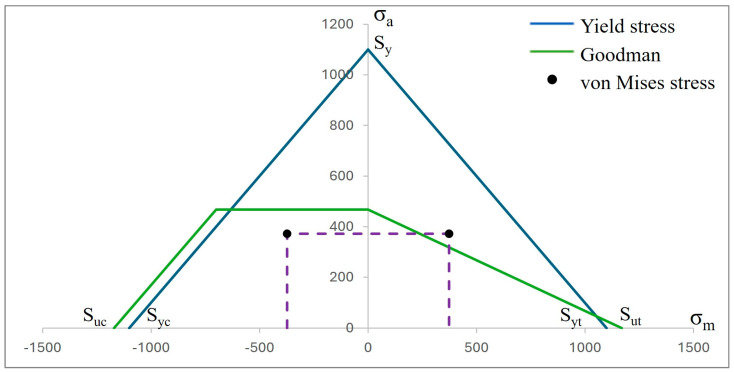
Graph σ_a_ vs. σ_m_ of fatigue behavior by Goodman’s theory for a lifetime of 1 × 10^6^ cycles of titanium (Ti_6_Al_4_V).

**Figure 14:**
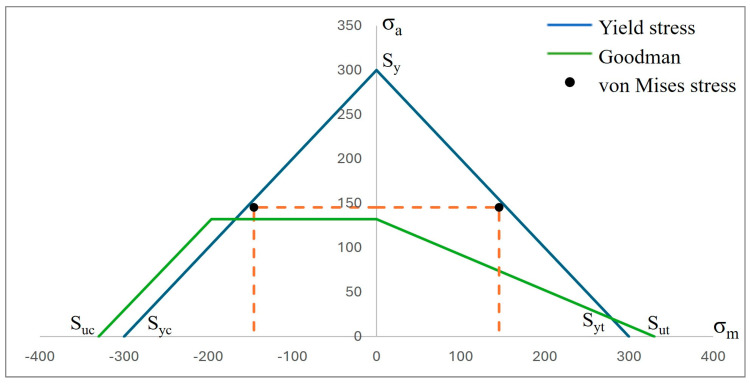
Graph σ_a_ vs. σ_m_ of fatigue behavior by Goodman’s theory for a life of 1 × 10^6^ cycles of polyether–ether–ketone (CFR-PEEK).

Complementing the above results, two graphs are presented where the fatigue behavior of both biomaterials can be observed (Figure 13 and Figure 14). This is with respect to the following two failure criteria: one is by Godman and the other is the fatigue strength criterion for negative mean stresses. In this sense, as mentioned above, titanium, due to its high rigidity, proved to be more resistant, although CFR-PEEK showed better mechanical behavior because of the fact that the value of the alternating stress is lower than that of titanium. However, the safety factor is below unity, with a value of 0.90 with respect to the value of 1.25 of titanium, which clearly indicates that the polymer will fail.

## 4. Discussion

The results obtained from the numerical simulation allow us to observe the biomechanical behavior of a dental implant under the application or presence of external forces, in this case, masticatory forces. Such is the case of general displacements, where it can be clearly seen that the maximum movement is in the upper area of the pillar, and derived from this movement, an elastic deformation occurred. In the case of titanium, this deformation occurred in the bone where it came into contact with the implant. The opposite was the case for CFR-PEEK, in which the deformation manifested itself in the implant in the root thread area. This aspect is related to the stiffness of both biomaterials and indicates, from a biomechanical point of view, how the distribution of forces behaves along the structure. In addition to the above, and taking as a reference the von Mises fault criterion, which shows the area most susceptible to failure, in the case of titanium, the dental implant would tend to fail in the thread area of the dental implant body, more specifically, at the root of the thread, and, in the case of CFR-PEEK, the critical zone would be in the bone. This shows that with the polymer, there is a decrease in the effect of tension shielding because, being a biomaterial with a rigidity similar to that of bone, it tends to deform together with bone and transmit the load to it. In the case of titanium, being a harder material than bone itself, it prevents the implant from deforming, causing most of the effort to be concentrated on the implant. In addition, the critical area is at the root of the thread due to the fact that it is not a rope with acute angles but is rounded, and that causes that area to behave like a concentrator. Otherwise, it would behave as an intensifier of effort, causing a failure with less effort.

Consequently, the results obtained from the fatigue assessment indicate the number of load cycles required for the material to fail. In the case of titanium, based on the results of von Mises stress, it was compared with two fatigue failure criteria including Goodman’s and negative mean stresses. In the graphs presented in the Results Section, it can be clearly seen that the material fails based on Goodman’s fatigue failure criteria; however, this is not relevant to this case since the load cycles of the chewing force acting on the dental implant are not 100% tension–tension. The opposite situation occurs in the case of the fatigue failure criteria of negative average stresses, where there is a behavior of the load cycles in the form of compression–compression on the dental implant, and this is verified with the value of the alternating stress, which is 372.85 Mpa, which is below the fatigue limit of the material of 468 MPa, meaning that the dental implant will not fail and will have an “infinite” life (1 × 10^9^ life cycles). Now, the problem was solved with CFR-PEEK because the results indicate that based on both criteria of fatigue failure, the material tends to fail. Despite the fact that the maximum value of the load cycles was 573,780, it is considered within the range of high-load cycles. It is also important to remember that the parameters considered represent a bone resection, so it is very likely that in healthy conditions, the dental implant will not fail due to fatigue. For example, in the work by Ziaie and Mohammad [62], they present a fatigue analysis where the alternate effort is less than that of this research work; however, the difference is that the dental implant is placed to the bone by simulating healthy conditions. In other works, such as those by Hosseini-Faradonbeh et al. [63], De Stefano et al. [64], and Abdoli et al. [65], when performing experimental evaluations of fatigue tests in dental implants, it can be clearly observed that the critical failure with loading conditions occurs in the thread area of the dental implant body, which is the same result obtained in this work but by numerical evaluation. In addition, it should be noted that the works mentioned above are evaluations with titanium as a material, so this study complements the already-known parameters and integrates great future expectations to replace metallic materials with composite polymeric materials and with treatments that can provide equal or even better biological, physical, and mechanical conditions. However, although it is clear that the polymeric material will fail, an important aspect to consider is that it has viscoelastic properties, which could somehow harm or favor the biomechanical support of the dental implant. However, for this case study, the authors neglected this property of the material since the model is considered elastic, linear, and isotropic. However, considering the results of CFR-PEEK, it would be prudent to perform a nonlinear evaluation. Finally, this demonstrates the effectiveness and importance of the use and application of numerical methods for the evaluation of models and/or biomodels, no matter how complex they may be. This method can even impact various areas of study such as aeronautics, mechanics, biomedical, biomechanics, etc. By applying this method, experimental evaluation processes involving animals have been reduced, which optimizes time, money, and materials and offers assertive results with a high percentage of similarity to reality.

## 5. Conclusions

The results obtained from the static analysis under a single loading condition show the susceptible areas where the material tends to fail, although the magnitude of the values obtained from both materials never exceeded the yield strength. However, for conditions of multiple load cycles, CFR-PEEK exceeded the yield strength and fatigue limit, although it was minimal. In addition, as mentioned above, it would be necessary to carry out a non-linear analysis on both materials and, in the case of polymers, to observe the elastoplastic behavior due to its viscous property. In addition to the above, this numerical analysis showed that stiffness with properties more similar to those of bone does reduce the stress shielding effect. On the other hand, the designs of the dental abutments proved to be geometrically optimal because the variation in the results was minimal. For both materials, it could be safely said that due to static charge, it would not fail. Finally, despite the fact that CR-PEEK failed because of fatigue, its mechanical behavior and its physical and mechanical properties make it a promising biomaterial for dental applications, especially in the area of dental implantology. It is a biomaterial that, in the last 3 years, has had a growth in research, and the authors have no doubt that in the near future, the first tests will be carried out in patients.

## Figures and Tables

**Table 1:** Mechanical properties.

Material	Young’s Modulus (GPa)	Poisson’s Ratio	Density (Kg/m^3^)	Yield Strength (MPa)	Ultimate Strength (MPa)
Ti_6_Al_4_V	114	0.36	4430	1100	1170
ZrO_2_	210	0.31	6100	2000	900
CFR-PEEK	24	0.38	1400	300	330
Cortical bone *	E_x_ = 12.6	ν_xy_ = 0.3	1700	-	-
	E_y_ = 12.6	ν_yx_ = 0.3			
	E_z_ = 19.4	ν_yz_ = 0.253			
	G_xy_ = 4850	ν_zy_ = 0.39			
	G_yz_ = 5700	ν_xz_ = 0.253			
	G_xz_ = 5700	ν_zx_ = 0.39			
Trabecular bone *	E_x_ = 1.148	ν_xy_ = 0.055	270	-	-
	E_y_ = 2.70	ν_yx_ = 0.01			
	E_z_ = 1.148	ν_yz_ = 0.01			
	G_xy_ = 68	ν_zy_ = 0.055			
	G_yz_ = 68	ν_xz_ = 0.322			
	G_xz_ = 434	ν_zx_ = 0.055			

* These mechanical properties can vary from person to person, so approximate values were considered to be an apparently healthy person.

**Table 2:** Maximum and minimum results obtained for each case study.

Element		Total Displacement (mm)		von Mises Stress (MPa)		Fatigue Life (Cycles)	
		Titanium	CFR-PEEK	Titanium	CFR-PEEK	Titanium	CFR-PEEK
Snappy abutment	Max.	0.1108	0.4653	745.71	290.05	1 × 10^9^	5.73 × 10^5^
	Min.	0	0	0	0	1 × 10^9^	1 × 10^9^
Universal abutment	Max.	0.1156	0.4814	787.1	296.94	1 × 10^9^	5.10 × 10^5^
	Min.	0	0	0	0	1 × 10^9^	1 × 10^9^
Esthetic abutment	Max.	0.1161	0.4833	786.53	296.92	1 × 10^9^	5.10 × 10^5^
	Min.	0	0	0	0	1 × 10^9^	1 × 10^9^

## Data Availability

Data are contained within the article.

## References

[1] Chen X., Ma R., Min J., Li Z., Yu P., Yu H. (2020). Effect of PEEK and PTFE coatings in fatigue performance of dental implant retaining screw joint: An in vitro study. J. Mech. Behav. Biomed. Mater..

[2] Sun F., Wang L., Li X.C., Cheng W., Lin Z., Ba D.C., Song G.Q., Sun C.S. (2020). Effect of surface modification on the long-term stability of dental implant abutment screws by plasma nitriding treatment. Surf. Coat. Technol..

[3] Boehm M.W., Yakubov G.E., Stokes J.R., Baier S.K. (2020). The role of saliva in oral processing: Reconsidering the breakdown path paradigm. J. Texture Stud..

[4] Nicholson J.W. (2020). Titanium alloys for dental implants: A review. Prosthesis.

[5] Yamaguchi S., Yamanishi Y., Machado L.S., Matsumoto S., Tovar N., Coelho P.G., Thompson V.P., Imazato S. (2018). In vitro fatigue tests and in silico finite element analysis of dental implants with different fixture/abutment joint types using computer-aided design models. J. Prosthodont. Res..

[6] Yamanishi Y., Yamaguchi S., Imazato S., Nakano T., Yatani H. (2012). Influences of implant neck design and implant-abutment joint type on peri-implant bone stress and abutment micromovement: Three-dimensional finite element analysis. Dent. Mater..

[7] Scaizer Lopes G.R., Melo de Matos J.D., Queiroz D.A., Mendes Tribst J.P., Ramos N.C., Garcia Rocha M., Baldotto Barbosa A., Bottino M.A., Souto Borges A.L., Sussumu Nishioka R. (2022). Influence of abutment design on biomechanical behavior to support a screw-retained 3-unit fixed partial denture. Materials.

[8] Singh A., Singh A., Vivek R., Chaturvedi T.P., Chauhan P., Gupta S. (2013). SEM analysis and management of fracture dental implant. Case Rep. Dent..

[9] Vinhas A.S., Aroso C., Salazar F., López-Jarana P., Ríos-Santos J.V., Herrero-Climent M. (2020). Review of the mechanical behavior of different implant-abutment connections. Int. J. Environ. Res. Public Health.

[10] Prasad S., Ehrensberger M., Prasad Gibson M., Kim H., Monaco E.A. (2015). Biomaterial properties of titanium in dentistry. J. Oral Biosci..

[11] Gosavi S., Gosavi S., Alla R. (2013). Titanium: A miracle metal in dentistry. Trends Biomater. Artif. Organs.

[12] Messer R., Wataha J. (2002). Dental Materials: Biocompatibility. Encyclopedia of Materials: Science and Technology.

[13] Alrabeah G.O., Brett P., Knowles J.C., Petridis H. (2017). The effect of metal ions released from different dental implant-abutment couples on osteoblast function and secretion of bone resorbing mediators. J. Dent..

[14] Romero-Resendiz L., Gómez-Sáez P., Vicente-Escuder A., Amigó-Borrás V. (2021). Development of Ti-In alloys by powder metallurgy for application as dental biomaterial. J. Mater. Res. Technol..

[15] Kaur M., Singh K. (2019). Review on titanium and based alloys as biomaterials for orthopaedic applications. Mater. Sci. Eng. C.

[16] Xiong Y., Wang W., Gao R., Zhang H., Dong L., Qin J., Wang B., Jia W., Li X. (2020). Fatigue behavior and osseointegration of porous Ti-6Al-4V scaffolds with dense core for dental application. Mater. Des..

[17] Poggio C.E., Ercoli C., Rispoli L., Maiorana C., Esposito M. (2017). Metal-free materials for fixed prosthodontic restorations. Cochrane Database Syst. Rev..

[18] Hariram V. (2024). A Brief Review on PEEK as biomaterial, Importance of Implant Design, 3D Printing and FEA in Dental Implant. E3S Web Conf..

[19] AlOtaibi N., Naudi K., Conway D., Ayoub A. (2020). The current state of PEEK implant osseointegration and future perspectives: A systematic review. Eur. Cells Mater..

[20] Rokaya D., Srimaneepong V., Sapkota J., Qin J., Siraleartmukul K., Siriwongrungson V. (2018). Polymeric materials and films in dentistry: An overview. J. Adv. Res..

[21] Xu X., He L., Zhu B., Li J., Li J. (2017). Advances in polymeric materials for dental applications. Polym. Chem..

[22] Huang H.Y., Feng S.W., Chiang K.Y., Li Y.C., Peng T.Y., Nikawa H. (2023). Effects of various functional monomers’ reaction on the surface characteristics and bonding performance of polyetheretherketone. J. Prosthodont. Res..

[23] Alqurashi H., Khurshid Z., Yaqin Syed A.U., Rashid Habib S., Rokaya D., Zafar M.S. (2021). Polyetherketoneketone (PEEK): An emerging biomaterial for oral implants and dental prostheses. J. Adv. Res..

[24] Schwitalla A.D., Zimmermann T., Spinting T., Kallage I., Müller W.D. (2017). Fatigue limits of different PEEK materials for dental implants. J. Mech. Behav. Biomed. Mater..

[25] Sun C., Kang J., Yang C., Zheng J., Su Y., Dong E., Liu Y., Yao S., Shi C., Pang H. (2022). Additive Manufactured Polyether–Ether–Ketone Implants for Orthopaedic Applications: A Narrative Review. Biomater. Transl..

[26] Ma H., Suonan A., Zhou J., Yuan Q., Liu L., Zhao X., Lou X., Yang C., Li D., Zhang Y. (2021). gang PEEK (Polyether–Ether–Ketone) and Its Composite Materials in Orthopedic Implantation. Arab. J. Chem..

[27] Wang B., Huang M., Dang P., Xie J., Zhang X., Yan X. (2022). PEEK in Fixed Dental Prostheses: Application and Adhesion Improvement. Polymers.

[28] Shala K., Tmava-Dragusha A., Dula L., Pustina-Krasniqi T., Bicaj T., Ahmedi E., Lila Z. (2018). Evaluation of maximum bite force in patients with complete dentures. Open Access Maced. J. Med. Sci..

[29] Fontolliet A., Al-Haj Husain N., Özcan M. (2020). Wear analysis and topographical properties of monolithic zirconia and CoCr against human enamel after polishing and glazing procedures. J. Mech. Behav. Biomed. Mater..

[30] Shinya A. (2023). Dental material research in prosthodontics—Towards developing better and efficient biomimetic materials. J. Prosthodont. Res..

[31] Suphangul S., Rokaya D., Kanchanasobhana C., Rungsiyakull P., Chaijareenont P. (2022). PEEK Biomaterial in Long-Term Provisional Implant Restorations: A Review. J. Funct. Biomater..

[32] Souza J.C., Pinho S.S., Braz M.P., Silva F.S., Henriques B. (2021). Carbon fiber-reinforced PEEK in implant dentistry: A scoping review on the finite element method. Comput. Methods Biomech. Biomed. Eng..

[33] Altiparmak N., Polat S., Onat S. (2023). Finite Element Analysis of the biomechanical effects of titanium and CFR-PEEK additively manufactured subperiosteal jaw implant (AMSJI) on maxilla. J. Stomatol. Oral Maxillofac. Surg..

[34] Naghavi S.A., Lin C., Sun C., Tamaddon M., Basiouny M., Garcia-Souto P., Taylor S., Hua J., Li D., Wang L. (2022). Stress Shielding and Bone Resorption of Press-Fit Polyether–Ether–Ketone (PEEK) Hip Prosthesis: A Sawbone Model Study. Polymers.

[35] Verma V., Hazari P., Verma P. (2023). Do implants made of polyetheretherketone and its composites have reduced stress shielding effects compared to other dental implant materials? A systematic review. Evid.-Based Dent..

[36] Parra Reyes D. (2021). Systematic review of the literature on the evaluation of the chewing process. Areté.

[37] Ayllón J.M., Navarro C., Vázquez J., Domínguez J. (2014). Fatigue life estimation in dental implants. Eng. Fract. Mech..

[38] Slavicek G. (2010). Human mastication. Int. J. Stomatol. Occlusion Med..

[39] Kayabasi O., Yuzbasioglu E., Erzincanli F. (2006). Static, dynamic and fatigue behaviors of dental implant using finite element method. Adv. Eng. Softw..

[40] Voumard B., Maquer G., Heuberger P., Zysset P.K., Wolfram U. (2019). Peroperative estimation of bone quality and primary dental implant stability. J. Mech. Behav. Biomed. Mater..

[41] Steiner J.A., Ferguson S.J., Harry G. (2016). Screw insertion in trabecular bone causes peri-implant bone damage. Med. Eng. Phys..

[42] Gomes C., Mesnard M., Ramos A. (2023). Bone density and proximal support effects on dental implant stability—Finite Element Analysis and in vitro experiments. J. Stomatol. Oral Maxillofac. Surg..

[43] Falcinelli C., Valente F., Vasta M., Traini T. (2023). Finite element analysis in implant dentistry: State of the art and future directions. Dent. Mater..

[44] Shivakumar S., Kudagi V.S., Talwade P. (2021). Applications of finite element analysis in dentistry: A review. J. Int. Oral Health.

[45] Lisiak-Myszke M., Marciniak D., Bieliński M., Sobczak H., Garbacewicz Ł., Drogoszewska B. (2020). Application of Finite Element Analysis in Oral and Maxillofacial Surgery—A Literature Review. Materials.

[46] Vieira F.R., Bitencourt S.B., Rosa C.D.D.R.D., Vieira A.B., Dos Santos D.M., Goiato M.C. (2023). Influence of different restoring materials on stress distribution in prosthesis on implants: A review of finite element studies. Eur. J. Dent..

[47] Peña A., Gallardo E.A., Morán A., Bravo J.A., Moreno M., Vite M. (2013). Microabrasion on dental restorative porcelains and amalgam. Tribol.—Mater. Surf. Interfaces.

[48] Romanyk D.L., Vafaeian B., Addison O., Adeeb S. (2020). The use of finite element analysis in dentistry and orthodontics: Critical points for model development and interpreting results. Semin. Orthod..

[49] Zeno K.G., Ammoury M.J. (2023). The surge of finite element analysis in the study of orthodontic mechanics: Are the findings applicable in practice?. Semin. Orthod..

[50] Xu Y., Fan Y., Xu G. (2023). Progress of finite element analysis method for oral implantology. Acad. J. Med. Health Sci..

[51] Poli O., Manzon L., Niglio T., Ettorre E., Vozza I. (2021). Masticatory force in relation with age in subjects with full permanent dentition: A cross-sectional study. Healthcare.

[52] Hernández-Vázquez R.A., Romero-Ángeles B., Urriolagoitia-Sosa G., Vázquez-Feijoo J.A., Vázquez-López A.J., Urriolagoitia-Calderón G. (2018). Numerical análisis of masticatory forces on a lower first molar considering the contact between dental tissues. Appl. Bionics Biomech..

[53] da Hora Sales P.E., Pessoa Barros A.W., Barbosa de Oliveira-Neto O., Camello F.J., Tavares Carvalho A.A., Carneiro Leão J. (2023). Do zirconia dental implants present better clinical results than titanium dental implants? A systematic review and meta-analysis. J. Stomatol. Oral Maxillofac. Surg..

[54] Knapic D., Muck M., Heitz J., Baumgartner W., Mandare A.I., Kleber C., Hassel A.W. (2023). Electrochemical and surface characterization of anodized and fs-laser treated Ti6Al4V for osseo-repellent bone screws and dental implants. Electrochim. Acta.

[55] Ortega B., González H. (2021). Functionalization of polymeric prostheses by thermal spraying: A review. Rev. Colomb. Mater..

[56] Martinez-Mondragon M., Urriolagoitia-Sosa G., Romero-Ángeles B., Maya-Anaya D., Martínez-Reyes J., Gallegos-Funes F.J., Urriolagoitia-Calderón G.M. (2022). Numerical analysis of Zirconium and Titanium implants under the effect of critical masticatory load. Materials.

[57] Martinez-Mondragon M., Urriolagoitia-Sosa G., Romero-Ángeles B., Pérez-Partida J.C., Cruz-Olivares I.M., Urriolagoitia-Calderón G.M. (2023). Bilinear numerical analysis of the structural behavior of a dental implant applied as a biomaterial carbon fiber reinforced polyether-ether-ketone (CFR-PEEK): A finite element analysis. Dent. Hypotheses.

[58] Bachiri A., Djebbar N., Boutabout B., Serier B. (2020). Effect of different impactor designs on biomechanical behavior in the interface bone-implant: A comparative biomechanics study. Comput. Methods Programs Biomed..

[59] Figueroa-Hernández C., Pantaleón-Matamoros E., Méndez-González S., García-Fernández C., Gómez-González R., Carvajal-de la Osa J. (2020). Análisis del fenómeno de fatiga en implantes dentales monocomponente. Ing. Mecánica.

[60] Shemtov-Yona K., Rittel D. (2016). Fatigue of Dental Implants: Facts and Fallacies. Dent. J..

[61] García-González M., Blasón-González S., García-García I., Lamela-Rey M.J., Fernández-Canteli A., Álvarez-Arenal Á. (2020). Optimized Planning and Evaluation of Dental Implant Fatigue Testing: A Specific Software Application. Biology.

[62] Ziaie B., Khalili S.M.R. (2021). Evaluation of Fatigue Life for Dental Implants Using FEM Analysis. Prosthesis.

[63] Hosseini-Faradonbeh S.A., Katoozian H.R. (2022). Biomechanical evaluations of the long-term stability of dental implant using finite element modeling method: A systematic review. J. Adv. Prosthodont..

[64] De Stefano M., Lanza A., Sbordone L., Ruggiero A. (2023). Stress-strain and fatigue life numerical evaluation of two different dental implants considering isotropic and anisotropic human jaw. Proc. Inst. Mech. Eng. Part H J. Eng. Med..

[65] Abdoli Z., Mohammadi B., Karimi H.R. (2024). On the fatigue life of dental implants: Numerical and experimental investigation on configuration effect. Med. Eng. Phys..

